# Cultivating Affective Resilience: Proof-of-Principle Evidence of Translational Benefits From a Novel Cognitive-Emotional Training Intervention

**DOI:** 10.3389/fpsyg.2021.585536

**Published:** 2021-03-09

**Authors:** Sanda Dolcos, Yifan Hu, Christian Williams, Paul C. Bogdan, Kelly Hohl, Howard Berenbaum, Florin Dolcos

**Affiliations:** ^1^Department of Psychology, University of Illinois at Urbana–Champaign, Champaign, IL, United States; ^2^Beckman Institute for Advanced Science and Technology, University of Illinois at Urbana–Champaign, Champaign, IL, United States; ^3^Neuroscience Program, University of Illinois at Urbana–Champaign, Champaign, IL, United States

**Keywords:** emotion regulation training, transfer effects of intervention, affective resilience, emotional well-being, emotional memory recollection, emotion-cognition interactions, large-scale functional brain networks, resting state fMRI

## Abstract

Available evidence highlights the importance of emotion regulation (ER) in psychological well-being. However, translation of the beneficial effects of ER from laboratory to real-life remains scarce. Here, we present proof-of-principle evidence from a novel cognitive-emotional training intervention targeting the development of ER skills aimed at increasing resilience against emotional distress. This pilot intervention involved training military veterans over 5–8 weeks in applying two effective ER strategies [Focused Attention (FA) and Cognitive Reappraisal (CR)] to scenarios presenting emotional conflicts (constructed with both *external* and *internal* cues). Training was preceded and followed by neuropsychological, personality, and clinical assessments, and resting-state functional MRI data were also collected from a subsample of the participants. Results show enhanced executive function and psychological well-being following training, reflected in increased working memory (WM), post-traumatic growth (PTG), and general self-efficacy (GSE). Brain imaging results showed evidence of diminished bottom-up influences from emotional and perceptual brain regions, along with evidence of normalized functional connectivity in the large-scale functional networks following training. The latter was reflected in increased connectivity among cognitive and emotion control regions and across regions of self-referential and control networks. Overall, our results provide proof-of-concept evidence that resilience and well-being can be learned through ER training, and that training-related improvements manifested in both behavioral change and neuroplasticity can translate into real-life benefits.

## Introduction

The increasing prevalence of emotional disturbances, such as anxiety and depression, in the adult population is a major public health concern. It is estimated that by 2030 anxiety and depression will be among the most prevalent causes of disability worldwide. Because of their high prevalence (with over 40 million Americans having anxiety disorders alone), these emotional disturbances are associated with overwhelming long-term costs, which may affect the overall quality of life even decades later (Howard et al., 2014). Complicating the issue, these mental health problems often go untreated because the dominant models of delivering mental health treatment in clinical settings prevent many individuals from accessing such services (Kazdin, 2019). Moreover, deficient emotion regulation (ER), which is one of the core problems in emotional disorders, may persist even after treatment and remission, hence increasing the likelihood of relapse and the persistence of emotional distress (Kanske et al., 2012). Psychological resilience, which involves adaptive emotional responses in the face of adversity, can provide a protective buffer against the harmful effects of stressful events. Resilience is influenced by a combination of internal personal attributes and external factors (family and social networks support). Among the most important personal attributes associated with resilience is the ability to self-regulate emotional responses and adaptively engage cognitive/executive control. Here, we provide proof-of-concept evidence regarding the effectiveness of a novel training alternative that captures the complexity of ER in everyday life. Our training program is grounded in the *emotion regulation choice* framework (Sheppes et al., 2014) and is based on a multidimensional approach involving behavioral, personality, clinical, and brain imaging assessments.

Self-regulation influences the ability to control thoughts, emotions, and actions to achieve a desired outcome, and encompasses cognitive, emotional, neural, and behavioral levels (Mischel et al., 1989; Blair and Diamond, 2008). Deficient regulation of emotion (or emotion dysregulation) is one of the core problems in emotional disturbances, such as anxiety and depression, and may persist after therapeutic treatment and remission (Kanske et al., 2012). Self-regulation capacities indexing differences in vulnerability to anxiety and stress are related to both *psychological* and *neural* factors, highlighting the need to develop self-regulatory solutions based on an integrative understanding of psychological factors and their interactions, along with an understanding of the associated neural mechanisms. *Psychological* factors include both cognitive control [working memory (WM), inhibitory control] and ER skills (Naragon-Gainey et al., 2017), as also demonstrated by our own work (Hu and Dolcos, 2017; Moore et al., 2018). *Neural* factors include network-level (Power et al., 2011) targets for cognitive control (“*cognitive control networks*”), involving dorsolateral prefrontal cortical (dlPFC) and lateral parietal (LP) regions, and emotion/ER networks (“*salience/survival network*”), involving basic emotion processing regions, such as the amygdala (AMY), and regions of emotion integration and regulation, such as the ventrolateral PFC (vlPFC), medial PFC (mPFC), and anterior cingulate cortex (ACC; Hopfinger et al., 2000; Seeley et al., 2007; Dosenbach et al., 2008). The role of these networks in self-regulation has been identified based on both functional and structural neuroimaging studies. For instance, we have recently shown increased activity in the cognitive control network and reduced activity in regions of the salience network, when engaging specific ER strategies (Denkova et al., 2015; Iordan et al., 2019; Dolcos et al., 2020a). Also, increased gray matter volume in cognitive control regions provides protection against symptoms of anxiety (Dolcos et al., 2016; Hu and Dolcos, 2017; Moore et al., 2018).

Important to consider in this context are possible links between psychological and neural aspects in self-regulation, which highlight the importance of flexible adaptive behavior (Naragon-Gainey et al., 2017). For instance, increased focus on distressing thoughts and memories observed in affective disorders may be linked to an inability to flexibly switch attentional focus between internal and external environments, which may be the main cause for getting “*stuck in the rut*” (Nolen-Hoeksema, 1991; Cooney et al., 2010; Holtzheimer and Mayberg, 2011). This is consistent with evidence pointing to alterations in the *default* or resting-state functional connectivity (rsFC) of the large-scale brain networks linked to cognitive/executive and emotional dysfunctions, with increased coupling among regions on the *default mode network* (DMN) and functional decoupling of DMN from regions of the *frontoparietal control network* (FPCN; Hopfinger et al., 2000; Raichle et al., 2001; Fox et al., 2005; Dosenbach et al., 2008; Kaiser et al., 2015).

Recent cognitive neuroscience research offers some initial promising evidence that ER may be enhanced through cognitive training (Denny and Ochsner, 2014), although such evidence is limited. Cognitive training and interventions that train specific psychological abilities, such as mindfulness-based attention and multitasking performance, can induce changes in brain structure and function (Denny and Ochsner, 2014; Verghese et al., 2016; Valk et al., 2017; Cohen and Ochsner, 2018; Dolcos et al., 2020c). Evidence from these interventions complements clinical studies showing volume reductions in specific brain regions following traumatic life events (Sekiguchi et al., 2015), and provide evidence for possible enhancements induced by customized cognitive training. However, most of the work on ER training is limited because it has focused on practicing one specific strategy (Sekiguchi et al., 2015; Cohen and Ochsner, 2018; Dolcos et al., 2020c). In our view, such a “*one size fits all*” approach minimizes the complexity of ER, which inherently involves interactions of person, situation, and ER strategy factors (Aldao et al., 2015).

Our view is consistent with the *emotion regulation choice* framework, which proposes that ER involves two major cognitive stages, an attentional selection stage and a semantic meaning stage (Sheppes et al., 2014). The initial attentional stage consists of an early disengagement from emotional information before it undergoes elaborated processing. This is typically achieved through attentional deployment strategies, such as focused attention (FA), which involve disengaging attention from the emotional information before it is represented in working memory by focusing on neutral thoughts or details (Van Dillen and Koole, 2007). The subsequent semantic meaning stage involves later engagement with the emotional information that passes the early attentional selection stage (Sheppes et al., 2014). The most common semantic strategy is cognitive reappraisal (CR), which involves engaging with and modifying the meaning of emotional information through semantic processing (e.g., Gross, 2002). These early vs. later strategies have differential benefits. Specifically, blocking emotional information early, through attentional deployment, before it gathers force, allows modulation of high-intensity emotional information, by engaging relatively simple cognitive processes, whereas the elaborated semantic processing that occurs during CR allows processing, evaluating, and remembering emotional information, which are crucial for long-term goals and for adaptation (Sheppes et al., 2014). Psychological resilience and well-being require flexible adaptation of ER strategies to fit differing situational demands (Gross, 2007; Kashdan and Rottenberg, 2010; Watkins, 2011). Therefore, it is important to examine interactive effects of regulation strategies that are most beneficial for a given person in a given situation.

The following additional concepts are also central to consider in this context: *self-efficacy* (SE), *cognitive flexibility* (CF), and *working memory* (WM). The concept of SE, originating in the Social Cognitive Theory (SCT; Bandura, 1986), refers to individuals’ beliefs about their own competence to exert control over events that matter (Bandura, 1997). Such beliefs influence cognitive, affective, motivational, and decisional processes that support individuals in achieving their goals and are crucial determinants of initiating and maintaining changes in behavior (Bandura, 1986). Individuals with high SE engage in more effortful, persistent, and resilient coping efforts, which may enable them to identify important opportunities within stressful circumstances and promote individual growth (Bandura, 1997, 2001). Importantly, recent evidence identifying SE as a mechanism influencing the effects of stress on mental health, along with suggestions regarding the malleability and transfer of SE beliefs across functional domains, point to the importance of considering SE as a key concept in interventions aimed at improving mental health (Bandura, 1986; Maciejewski et al., 2000; Zinken et al., 2008; Schönfeld et al., 2019).

Regarding CF, given the complexity and shifting nature of contextual demands in everyday life, individuals may need to flexibly engage multiple regulation strategies, both within and across emotional episodes (ER flexibility; Kashdan and Rottenberg, 2010; Aldao et al., 2015; Ford et al., 2019). A rich repertoire of ER strategies, along with their flexible implementation linked to current contextual demands, have been associated with enhanced adaptation and better coping (Cheng, 2001; Bonanno et al., 2004; Kashdan and Rottenberg, 2010; Bonanno and Burton, 2013; Aldao et al., 2015; Koole et al., 2015; Levy-Gigi et al., 2016). Inflexibility, on the other hand, characterized by rigid attempts to control psychological reactions to discomfort, has been associated with increased distress and detrimental effects on self-efficacy (Gamez et al., 2014; Levin et al., 2014; Jeffords et al., 2018; Tavakoli et al., 2019).

Flexible engagement of ER strategies is also an important predictor of post-traumatic growth (PTG). PTG theory suggests that growth occurs by developing regulation strategies that encourage constructive thinking and allow individuals to engage with trauma-related emotions and memories (Tedeschi and Calhoun, 2004). Reappraisal, a strategy focused on engaging with and changing the meaning of the emotional content, has been consistently associated with PTG, but recent ER research supports a more nuanced, context-dependent view. For instance, reappraisal seems to be preferred when dealing with low-intensity traumatic events, and attention focus/distraction is preferred for coping with high-intensity distressing emotions (Sheppes et al., 2014; Orejuela-Dávila et al., 2019). Moreover, the use of reappraisal or distraction at different phases in the trauma recovery may have different effects, reflected in increased PTG or reduced post-traumatic stress (Levy-Gigi et al., 2016).

Finally, WM also plays a critical role in the ability to successfully engage ER and reduce symptoms of distress (Schmeichel and Demaree, 2010). Defined as the capacity-limited resource that temporally maintains and manipulates information in the service of higher functions, WM is an executive function that plays a key role in the regulation of cognitive and emotional processes at both early and later stages of processing (Baddeley, 2003). Evidence linking WM to the early stage control of visual attention shows that WM capacity contributed to the ability to focus one’s eyes away from a salient visual stimulus, and predicted the ability to ignore the “unattended” message in a dichotic-listening task (Conway et al., 2001; Kane et al., 2001). Moreover, WM capacity has been linked to the ability to engage later-stage ER strategies, such as reappraisal and suppression (Schmeichel et al., 2008; McRae et al., 2012). Finally, increased WM capacity is essential in reducing the detrimental impact of apprehensive thoughts, which tend to be “permanent residents” in the WM of anxious individuals (Eysenck et al., 2007).

The main goal of the present study was to investigate the effectiveness of a comprehensive cognitive-emotional training program aimed at developing healthy and flexible ER skills to increase resilience and well-being and improve executive function. This training program builds upon evidence, including from our own research, regarding cognitive and emotional control and their link to the associated neural mechanisms, and capitalizes on evidence regarding the effectiveness of two ER strategies in reducing emotional distress: focused attention (FA) and cognitive reappraisal (CR; Ochsner and Gross, 2005). This combination of strategies is based on evidence regarding their effectiveness in both healthy functioning and in clinical conditions, which allows for optimal adaptive responses when facing real-life emotional challenges (Kanske et al., 2012; Denkova et al., 2015; Iordan et al., 2019; Dolcos et al., 2020a,b). For instance, FA can be quickly deployed when individuals may unexpectedly encounter highly emotional stimuli (earlier stages of emotion processing) and CR can draw on a combination of cognitive control processes (later stages of emotion processing) to change one’s emotional appraisals and responses (Gross, 1998). Also, both strategies can be applied to *external* (percepts) and to *internal* (memories, thoughts) stimuli, as well as to developing problem-solving skills. Hence, these two aspects (i.e., external vs. internal) were both trained using a picture processing task and a writing task, respectively. Of particular relevance is also evidence regarding their effectiveness in influencing emotional memories, which is a topic less explored in the ER literature. For instance, we recently demonstrated the effectiveness of FA during both encoding of memories for emotional pictures and retrieval of emotional autobiographical memories (Denkova et al., 2015; Iordan et al., 2019; Dolcos et al., 2020a).

The effectiveness of the training was assessed using neuropsychological, personality, and clinical assessments, along with measures of rsFC, before and after the training. We tested the following hypotheses regarding the neurobehavioral effects of the present ER intervention: (1) behavioral improvements following training would be reflected in measures of both cognitive/executive and affective domains, and possibly in measures indexing more general abilities, such as self-efficacy, and positive psychological growth; (2) regarding brain imaging, we expected normalization of the rsFC in the large-scale functional networks following training, possibly reflected in decreased connectivity among DMN regions and increased connectivity between DMN and FPCN regions. We also explored evidence for diminished bottom-up influences, possibly linked to decreased rsFC of perceptual regions and AMY, as well as possible changes in the connectivity between basic emotion processing regions (AMY) and emotion control (PFC) regions.

## Materials and Methods

### Participants

Nineteen military veterans (with a deployment assignment to Iraq and/or Afghanistan in the preceding 5 years) enrolled in post-secondary education at the time of the study (mean age = 30.9; *SD* = 7.9; 95% males, 79% White/European American, 11% Black/African American, 5% Asian/Asian-American, and 5% Other) were recruited to participate in the present pilot intervention. Participants were assigned to one of two training programs: (1) a cognitive-emotional regulation training (CERT) program (*N* = 9); or (2) a psychosocial training (PSYCT) program (*N* = 10), ensuring equal proportions of participants with posttraumatic stress disorder (PTSD) – i.e., scores 33 or above on the PTSD Checklist – PCL-5 in each condition (Weathers et al., 2013). Branch of service was also considered when assigning participants to the two programs. All participants provided written informed consent under a protocol approved by the Institutional Review Board (IRB) of the University of Illinois at Urbana-Champaign.

### General Procedures

**Figure 1:**
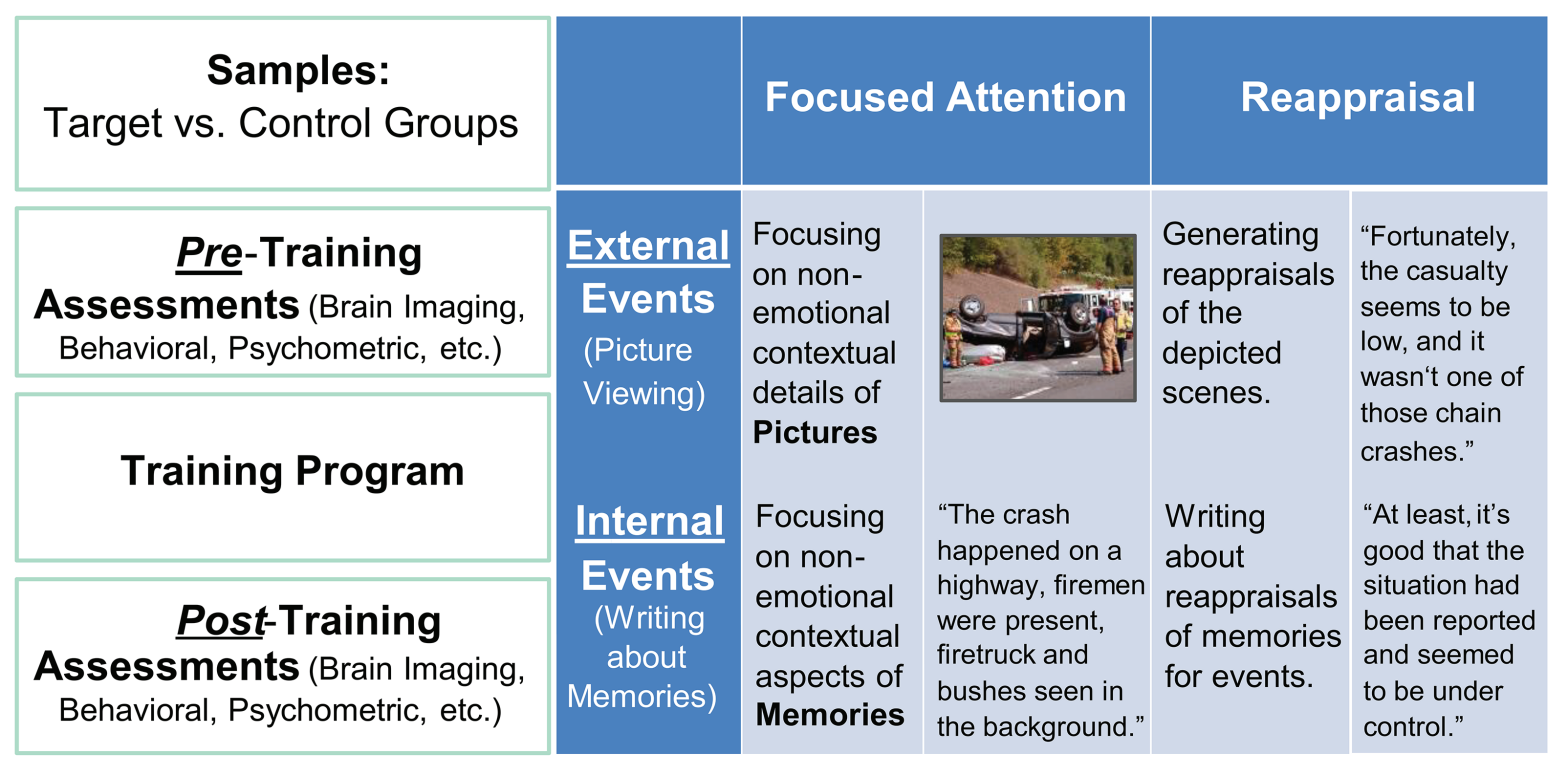
Illustration of the Intervention Approach. Big-picture details (left-side panel) and specific details regarding the implementation of ER training in the CERT group (right-side panel).

The intervention took place over 5–8 weeks, and participants in both conditions received equal amount of training/therapy per week (90 min), over two 45-min sessions for the CERT intervention and all in one session for the PSYCT intervention. The CERT participants had individual sessions led by an advanced doctoral student in cognitive neuroscience, where they learned how to apply the two ER strategies (i.e., FA and CR) to hypothetical scenarios (see Figure 1 below) in computerized tasks, and the PSYCT participants had group sessions with an advanced doctoral candidate in clinical psychology. All participants completed a set of self-report measures and a battery of cognitive tasks before and after the training. Ten participants (five from each group) also received structural and resting-state functional brain scans before and after the intervention.

### Training/Intervention Procedures

#### CERT Group

Participants in this group were individually guided by the researcher to perform computer-based tasks that simulated situations that required ER (Figure 1). Specifically, two types of situations were involved: external situations, where emotional challenges would arise from negative events in the surrounding environment, and internal situations, where emotional challenges would come from intruding negative autobiographical memories. The external ER was simulated using a picture-viewing task, where participants were instructed to apply ER strategies while viewing emotionally disturbing images. The internal ER was simulated using a timed writing task, where they were instructed to write about pre-identified emotional episodes from their autobiographical memories, in ways consistent with ER strategies. The autobiographical memories were identified using established procedures that have been effectively employed in our previous research to collect emotional autobiographical memories (Denkova et al., 2011, 2012, 2013a,b, 2015; Iordan et al., 2019). In the present study, participants were prompted with cues of a wide range of life events and were instructed to select and provide short descriptions of 12 most negative and 12 most positive events that reminded them of specific, unique, and personal events that they would like to work on during the training. Training primarily focused on the identified negative events. Due to the potential sensitive nature of past experiences of our subject population, a highly personalized approach was adopted, where participants were encouraged to identify six unpleasant episodes that they were comfortable working with on a regular basis, throughout the duration of the training. Because of the highly individualized nature of these memories, participants varied in the way they rated the emotional intensity associated with their own unpleasant memories, but overall they rated them as intense (mean intensity/arousal = 5.57; *SD* = 1.35) on a seven-point Likert scale (1 = “*Not at all*,” 7 = “*Very much*”). In each session, participants were guided to confront the presented emotional challenges, and practiced the application of both FA and CR to reduce the emotional impact created by the tasks.

In order to facilitate mastery over the strategies and encourage transfer effects to occur, participants were trained to switch between FA and CR, between the two types of tasks (involving internal thoughts and memories and external stimuli) and three temporal dimensions (present, past memories, and future worries). Several gradients were built into the session schedules. The training started out by familiarizing participants with one strategy at a time in a session, focusing on guiding participants to apply FA or CR to one internal and one external event selected by the participant. As the training advanced, each session posed increasingly complex situational demands, by guiding participants to first apply the same strategy to different events and eventually deploy different strategies on different events within a session, thereby achieving an increasing level of flexibility. Flexibility training culminated with sessions that engaged both strategies to work with worries for expected future events.

#### PSYCT Group

This intervention was developed by two of the authors (CW and HB), who are clinical psychologists and one of them (CW) is a military veteran. The Control intervention focused on the provision of skills (e.g., goal setting, problem solving) that were considered likely to be useful to military student veterans. This intervention emphasized skills that student veterans had likely already acquired in the military (e.g., time management) and explored how the skillset could be translated to an academic setting. Skills were presented and practiced in a group format, with participants given assignments to practice the skills between sessions. The intervention included elements of several evidence-based psychological treatments that were considered likely to be useful to military student veterans. Specifically, the intervention included elements of problem-solving therapy (Nezu, 2004), acceptance and commitment therapy (Hayes et al., 2006), cognitive therapy for depression (Beck, 1979), social skills training (Bellack et al., 1981), and behavioral activation treatment (Cuijpers et al., 2007). The treatment was provided in a group format (with group sizes ranging from 4 to 8), once per week for 90 min. Each week focused on a different aspect of psychoeducation and/or skill development (e.g., identifying values and cognitive distortions, clarifying goals, expressing anger, and recognizing thoughts, sensations, and behaviors associated with emotions).

### Symptom, Personality, and Neuropsychological Assessments

Posttraumatic Stress Disorder status was assessed using the PCL-5 (Weathers et al., 2013), which consists of 20 items that present challenges associated with a stressful experience (e.g., “*Repeated, disturbing dreams of the stressful experience*?”). Participants are instructed to report how much they are bothered by these challenges using a five-point scale (0 = “*Not at all*”; 4 = “*Extremely*”). An overall PCL-5 score is computed by summing the item ratings.

Anhedonic Depression was assessed using an abbreviated version of the Mood and Anxiety Symptom Questionnaire – Anhedonic Depression Scale (MASQ-AD; Bredemeier et al., 2010), which contains eight items asking participants how often they feel various positive and negative experiences (e.g., “*Felt really bored*”; “*Felt unattractive*”). Participants responded using a five-point scale, corresponding to how frequently they have experienced a variety of different symptoms during the past week (1 = “*Not at all*”; 5 = “*Extremely*”). Item ratings were summed to calculate the final MASQ-AD score. Some versions of this questionnaire ask participants how often they experience thoughts of suicide, but this question was omitted from our presentation due to IRB restrictions.

Trait Worry was assessed using an abbreviated version of the Penn State Worry Questionnaire (PSWQ; Meyer et al., 1990; Kertz et al., 2014), which consists of eight items, each presenting a statement concerning how often participants worry (e.g., “*I do not tend to worry about things*”). To each item, participants responded using a five-point scale, describing how applicable the statement was to them (1 = “*Not at all typical of me*”; 5 = “*Very typical of me*”). Item ratings were summed to calculate the final PSWQ scores.

Trait Affect was assessed using the Positive and Negative Affective Schedule (PANAS; Watson et al., 1988), which includes a list of 20 adjective descriptors of 10 positive (e.g., “*interested*,” “*enthusiastic*”) and 10 negative (e.g., “*irritable*,” “*upset*”) affects. Items were rated on a five-point scale (1 = “*Very slightly or not at all*”; 5 = “*Extremely*”) according to the extent to which “[the person] *feels this way over a longer period of time*.” This version of the PANAS was supplemented by adding three positive affect words (“*pleased*,” “*cheerful*,” and “*happy*”) and five negative affect words (“*frustrated*,” “*down*,” “*anxious*,” “*grouchy*,” and “*sad*”), for a total of 28 items (Boden et al., 2012). Positive and negative affect scores were summed for affect totals.

Post-Traumatic Growth was assessed using the PTG Inventory (PTGI; Tedeschi and Calhoun, 1996), which consists of 21 items that assess changes in peoples’ lives following a crisis or disaster. Each item presents participants a statement describing a potentially positive change in one’s mental state linked to the crisis (e.g., “*I can better appreciate each day*”; “*I changed my priorities about what is important in life*”). Participants rated the extent that they believed the statement applied to them, using a six-point scale (0 = “*I did not experience this change as a result of my crisis*”; 5 = “*I experienced this change to a very great degree as a result of my crisis*”). An overall PTGI score was created by summing the item ratings.

General Self-Efficacy was assessed using the General Self-Efficacy scale (GSE; Schwarzer and Jerusalem, 1995), which consists of 10 items assessing participants’ optimism and self-belief concerning difficult tasks and overcoming adversity. Participants were presented statements addressing how they tend to solve problems (e.g., “*When I am confronted with a problem, I can usually find several solutions*”) and were instructed to respond using a four-point scale corresponding to how much they believe the statement applies to them (1 = “*Not at all true*”; 4 = “*Exactly true*”). An overall GSE score was tallied by summing the values for each item.

Emotional Approach Coping was assessed using the Emotional Approach Coping scale (EAC; Stanton et al., 1994), which is an eight-item questionnaire designed to measure emotional coping. It contains two subscales, one on emotional processing (EP), addressing the extent that participants process their emotions in a healthy manner (e.g., “*I acknowledge my emotions*”), and a second subscale on emotional expression (EE), addressing whether participants are comfortable expressing their emotions (e.g., “*I let my feelings come out freely*”). For questions on each subscale, participants indicate how often they engage their emotions in these manners (1 = “*I usually do not do this at all*”; 4 = “*I usually do this a lot*”). Final EP and EE scores are calculated by summing the item ratings associated with each subscale. A total EAC score, reflecting the ability to acknowledge, understand, and express emotions, is also calculated, with lower scores representing poorer emotional coping.

Working memory was assessed using two versions of the Two-Back Working Memory task, one focusing on the spatial locations of the letter stimuli (Two-Back Space) and the other focusing on the identity of the letter stimuli (Two-Back Letter; Bredemeier and Berenbaum, 2013). For each version, participants were presented with a series of letter stimuli appearing at different spatial locations on the computer screen, one at a time, and responded *via* keyboard. Participants were instructed to maintain the two prior stimuli in their working memory. For the spatial version of the task, participants indicated whether the current stimulus was in the same or different location as the one presented two trials ago. For the letter version, participants responded to whether the current stimulus was the same as or different from the one presented two trials ago. Each stimulus was presented for 500 ms (with a 2,000 ms intertrial interval), and each participant completed five blocks of 20 stimuli for both space and letter tasks. Working memory performance was assessed as the percent accuracy and the average response time (RT) to correct trials. The first two trials were excluded from analyses, as they lack prior stimuli for comparison.

Attention and Executive Functions were measured using the Stroop test (Stroop, 1935). In this task, participants named the color of words while ignoring the content of the words (i.e., color words). The words used were all color words (e.g., red, blue, yellow, and green). The test takes advantage of individuals’ abilities to read words more quickly and automatically than naming colors.

### Brain Imaging Data Acquisition

MRI scanning was conducted on a 3T Siemens PRISMA scanner. Sagittal localizer and 3-D MPRAGE anatomical images were first obtained (TR = 2,300 ms; TE = 2.32 ms; FOV = 230 × 230 mm; volume size = 192 slices; voxel size = 0.9 × 0.9 × 0.9 mm^3^). Functional images consisted of a series of axial images acquired using an echoplanar sequence (TR = 2,000 ms; TE = 25 ms; FOV = 230 × 230 mm; volume size = 38 slices; voxel size = 2.5 × 2.5 × 3 mm^3^, number of volumes = 298), while participants stayed eyes-open.

### Data Analyses

#### Behavioral Data Analyses

The effects of interventions on cognitive and affective well-being were assessed by measuring participants’ pre- vs. post-intervention scores on the neuropsychological and questionnaire assessments. For each assessment, the simple main effects of the CERT and PSYCT training were first assessed by submitting participants’ scores to paired *t*-tests, done separately for each training group. Next, to compare the pre vs. post differences between the two groups, two-way mixed-ANOVAs were performed, with Time (Pre vs. Post) as the within-subject factor, and Group (CERT vs. PSYCT) as the across-subject factor. All the behavioral data were analyzed using the SPSS software (IBM Corp. 2017. version 25.0).

#### Brain Imaging Data Analyses

##### Preprocessing

Functional images collected at both timepoints (i.e., pre- and post-intervention) were despiked using 3dDespike in AFNI (Cox, 1996), before being submitted to preprocessing in SPM12,^1^ where they first underwent slice timing and two-pass realignment. The resulting motion parameters were later used to calculate scan-to-scan head displacement used for denoising. Co-registration was done in two steps: first between functional and anatomical images within each session, then between all images collected pre- and post- intervention. To take advantage of the repeated scans, the Longitudinal Registration toolbox (Ashburner and Ridgway, 2013) in SPM12 was used to first create mid-point average anatomical images for each participant, which were then segmented and submitted to groupwise Dartel template creation (Ashburner, 2007). Using this Dartel template, functional images were resampled to the MNI space on an isotropic 3 mm grid combining transformations estimated at previous steps in a single interpolation. Lastly, images were smoothed with a 6 mm FWHM Gaussian filter. Notably, smoothed data were used to define voxel time series at each voxel, whereas unsmoothed data were used to generate tissue type regressors used during denoising as well as to define seed timeseries for the chosen Regions of Interest (ROIs). After preprocessing, images were processed through denoising steps to further remove noises and artifacts, including (1) demeaning and detrending across each session, (2) nuisance regressions, which used a combination of motion regressors (six realignment parameters and their first-order derivatives), aCompCor (Behzadi et al., 2007) regressors (signals from top five principal components generated from each of the tissue maps of white matter and cortico-spinal fluids, along with their first-order derivatives), and main condition effects (computed by convolving images in a session with a canonical hemodynamic response function to further remove simple session-related co-activation confounds), (3) a simultaneous (Hallquist et al., 2013) bandpass filter of (0.008, 0.09) Hz, and (4) scrubbing based on framewise displacement (Power et al., 2011) with a threshold of 0.5 mm. Denoising steps were implemented in the CONN toolbox (v18b; Whitfield-Gabrieli and Nieto-Castanon, 2012). Denoised images were visually inspected to ensure the effectiveness of the procedures by observing the normality of the functional connectivity distributions, the relative independence between functional connectivity values and nodal distances, and that there were no substantial differences between the two experimental groups.

##### Functional Connectivity Analysis

A seed-based approach was implemented to examine the effects of interventions on the rsFC, based on clear a priory interest in regions involved in bottom-up emotion processing (i.e., amygdala, AMY) and top-down cognitive/emotional control processing, as follows: dorsolateral PFC (dlPFC), ventrolateral PFC (vlPFC), and medial PFC (mPFC; Moore et al., 2018). Two complementary atlases were used to define the masks of the seeds: the anatomically-based FSL Harvard-Oxford atlas (Frazier et al., 2005; Desikan et al., 2006; Makris et al., 2006; Goldstein et al., 2007) was used to define the seed mask for the subcortical structure, AMY, and the CONN network atlas (derived from ICA analyses of 497 subjects in a dataset from the Human Connectome Project) was used to define the seed masks for the cortical regions, with the lateral prefrontal cortex (PFC) node in the Frontoparietal network roughly corresponding to the dlPFC, the inferior frontal gyrus node in the Language network roughly corresponding to the vlPFC, and the Default Mode network node in the medial PFC, located anterior and ventral to the rostral ACC, roughly corresponding to the mPFC.

Seed timeseries were extracted from the unsmoothed data aggregated across all voxels within each seed ROI, and voxel timeseries were extracted from the smoothed data at each voxel in the rest of the brain. Functional connectivity values were calculated as the Pearson correlation coefficients between the selected ROI seeds and voxels in the rest of the brain, which were then Fisher-transformed into *z*-scores to allow subsequent statistical testing. At the second level, analyses focused on investigating Group × Time interaction effects, where differential functional connectivity changes were identified in the two groups as a result of the intervention, as well as a main effect of Time, where functional connectivity changes were identified across both groups. To this end, a general linear model was constructed, with Time being the within-subject factor and Group being the between-subject factor, each with two levels. Interaction effects were tested using the contrast [CERT(Post-Pre) − PSYCT(Post-Pre)], and the main effects contrast [CERT(Post-Pre) + PSYCT(Post-Pre)]. Unless otherwise specified, statistical significance was set at a voxel-level threshold of *p* < 0.005 (uncorrected) combined with a cluster-level threshold of *p* < 0.05 (FDR-corrected). These analyses were performed in the CONN toolbox (Whitfield-Gabrieli and Nieto-Castanon, 2012).

## Results

### Evidence of Enhanced Well-Being and Executive Function Following ER Training

**Figure 2:**
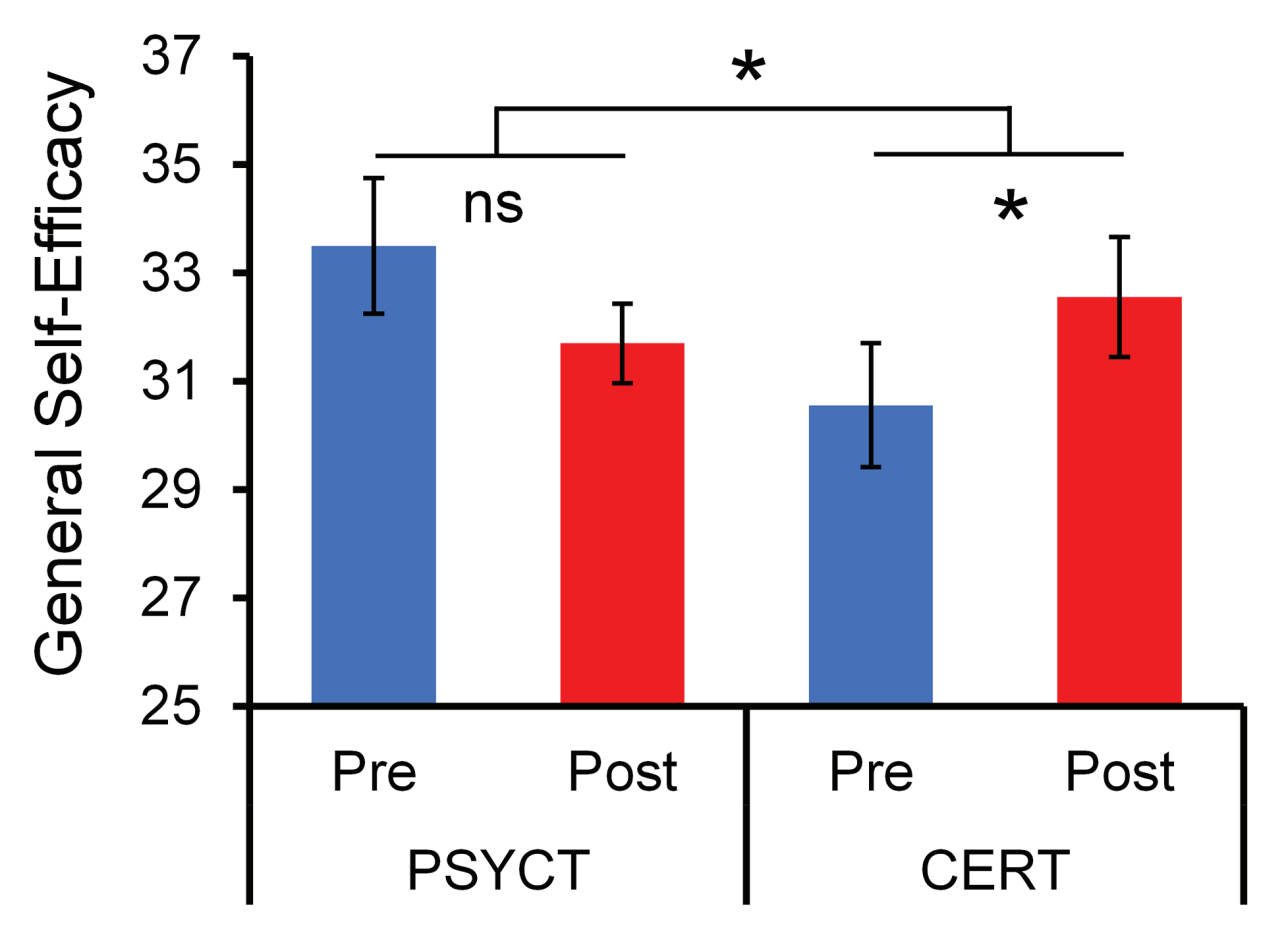
Increased General Self-Efficacy (GSE) following Cognitive – Emotional Regulation Training (CERT). Paired *t*-tests revealed a significant pre- to post-training increase in the GSE score that was specific to the CERT group (*N* = 9). This was confirmed by a two-way ANOVA Time (Pre vs. Post) × Group (PSYCT vs. CERT) interaction (*N* = 19). ^*^significant at *p* < 0.05 (two-tailed); ns, non-significant.

Table 1 summarizes the means and SDs of behavioral measures. As hypothesized, we identified behavioral patterns of increased abilities associated with the CERT training within domains relevant to adaptive cognitive and emotional processing. Specifically, the CERT group showed significant pre vs. post increases in GSE [*t*(8) = 2.910, *p* = 0.020, *d* = 0.970; Figure 2]. Additionally, participation in the CERT group promoted positive growth-focused mindsets with regard to participants’ traumatic events [PTGI: *t*(8) = 1.994, *p* = 0.041, one-tailed, *d* = 0.665]. While both changes support the efficacy of CERT training, only the benefits in GSE were found to be specific to the CERT group. This was demonstrated by a two-way mixed-design ANOVA examining the effect of Time (Pre vs. Post) and Training Group (CERT vs. PSYCT), which revealed a significant Time × Training Group interaction effect [*F*(1,17) = 7.196, *p* = 0.016, *η*_p_^2^ = 0.297], further supporting the effectiveness of our training in increasing self-efficacy and positive psychological change.

**Table 1:** Descriptive statistics linked to each intervention.

	PSYCT		CERT	
	Pre	Post	Pre	Post
**Measures:**				
PTSD PCL-5	22.4 (12.9)	18.7 (15.2)	21.8 (11.9)	22.1 (17.0)
MASQ-AD	17.7 (6.4)	16.4 (7.2)	17.7 (6.0)	19.3 (6.6)
PSWQ	24.4 (8.8)	20.0 (10.0)^*^	23.1 (8.6)	22.7 (8.8)
PANAS-Pos	33.1 (7.1)	32.9 (7.8)	29.3 (9.5)	27.8 (8.2)
PANAS-Neg	14.6 (3.0)	13.7 (2.8)	15.9 (5.1)	15.9 (5.4)
PTGI	38.6 (27.8)	42.8 (30.1)	26.9 (24.6)	38.4 (25.3)^†^
EAC-Total	21.6 (4.6)	22.7 (5.9)	18.9 (4.0)	20.3 (6.7)
EAC-EP	11.7 (4.1)	12.6 (3.2)	10.1 (2.1)	11.2 (3.3)
EAC-EE	9.9 (3.5)	10.1 (3.6)	8.8 (3.2)	9.1 (3.2)
GSE	33.5 (4.0)	31.7 (2.3)	30.6 (3.4)	32.6 (3.3)^*^
2-Back Letter	1,022 (141)	939 (191)	1,111 (194)	921 (192)^*^
2-Back Space	865 (187)	840 (162)	921 (241)	775 (175)^*^
Stroop	87 (19)	52 (19)	66 (17)	81 (48)
**Demographics:**				
Age (yr.)	29.2 (1.5)		32.0 (4.3)	
Male (%)	100		89	
Ethnicity (%) White Black Asian Other	70 20 10 0		89 0 0 11	
Education (yr.)	13.8 (0.6)		13.9 (0.5)	

Values represent means (SDs). Two-Back values reflect reaction times (RTs) in milliseconds (ms), and Stroop values reflect RT differences (ms) between incongruent and congruent trials. Significance markings, ^†^ and ^*^, indicate significant pre vs. post differences. PSTD PCL, Posttraumatic Stress Disorder Checklist; MASQ-AD, Mood and Anxiety Symptom Questionnaire – Anhedonic Depression Scale; PSWQ, Penn State Worry Questionnaire; PANAS, Positive and Negative Affective Schedule (Pos, Positive subscale; Neg, Negative subscale); PTGI, Post Traumatic Growth Inventory; EAC, Emotional Approach Coping (EP, Emotional Processing subscale; EE, Emotional Expression subscale); GSE, General Self Efficacy.

* significant at *p* < 0.05 (two-tailed);

† significant at *p* < 0.05 (one-tailed).

**Figure 3:**
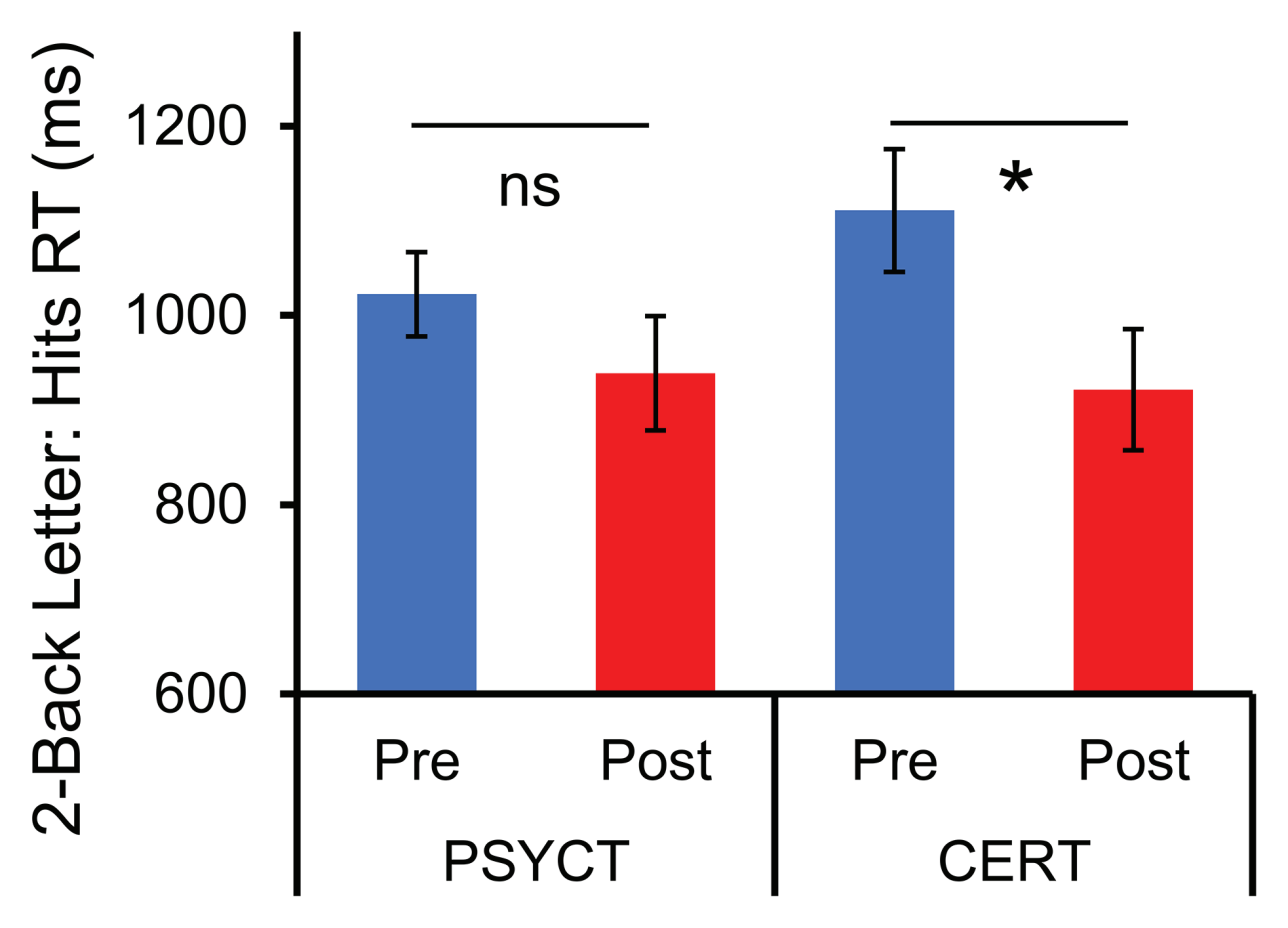
Increased Working Memory Performance following CERT Intervention. Figure shows the results of the Two-Back Letter task, and the same patterns were also observed for the Space task (not shown). Paired t-tests of pre- and post-training reaction times (RT) within the Two-Back WM tasks revealed significant improvements (faster time), which were exclusive to the CERT group. ^*^significant at *p* < 0.05 two-tailed; ns, non-significant.

Second, also as hypothesized, participants in the CERT group also showed post-training improvements in executive function (Figure 3), and these increases were observed in both Two-Back Letter and Space WM tasks. These findings were confirmed by paired samples *t*-tests for both the Two-Back Letter and Two-Back Space tasks, which both showed a decrease in reaction time in the CERT group [Letter: *t*(8) = −3.172, *p* = 0.013, *d* = −1.06; Space: *t*(8) = −2.535, *p* = 0.035, *d* = −0.845]. Although a two-way mixed-design ANOVA did not identify significant Time × Training Group interactions for either the Letter [*F*(1,17) = 1.38, *p* = 0.257, η_p_^2^ = 0.077] or Space [*F*(1,18) = 2.62, *p* = 0.122, η_p_^2^ = 0.146] tasks, the effect of training on WM was significant only in the CERT group, but not in the PSYCT group [Letter: *t*(9) = 1.279, *p* = 0.233, *d* = −0.404; Space: *t*(9) = 0.650, *p* = 0.532, *d* = −0.206]. These results indicate that improvements in executive domains were exclusive to the CERT training group. No other significant changes linked to the interventions were identified (all *p*_s_ > 0.05).

### Changes in Resting-State Functional Connectivity Following ER Training

**Figure 4:**
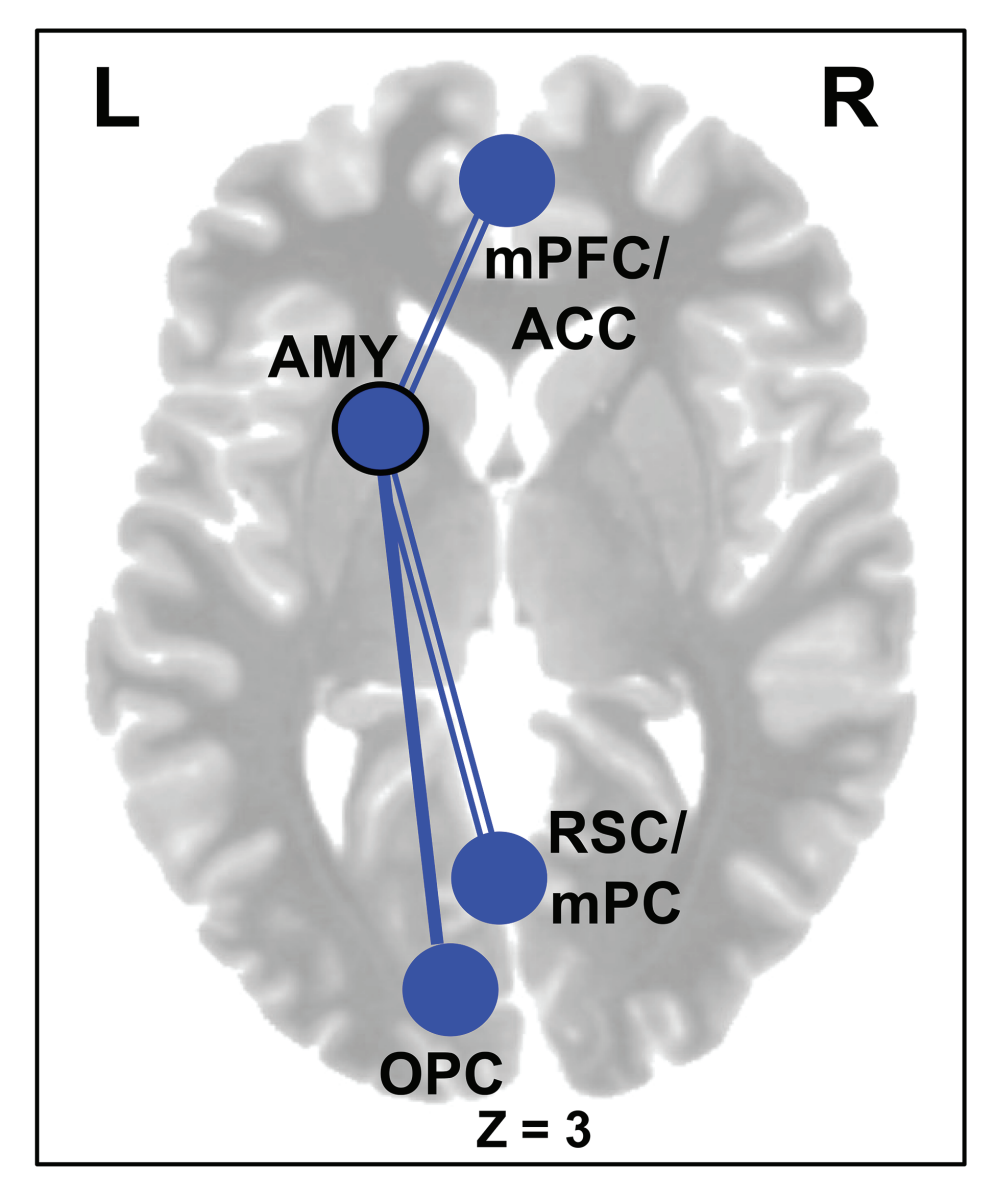
Decreased amygdala (AMY) rsFC following Training. Subjects across both CERT and PSYCT groups showed reductions in left AMY-medial prefrontal frontal cortex (mPFC) and left AMY-RSC/mPC rsFC following training (double lines); similar reduced rsFC was also identified for the right AMY (not shown), at a lower threshold (see Table 2). Interaction effects additionally revealed larger post-training reduction in rsFC between the left AMY seed and the left OPC, in the CERT group (single line; see also Table 2). Blue nodes and edges reflect decreases in rsFC. Black node outline indicates the seed region. mPFC, Medial Prefrontal Cortex; ACC, Anterior Cingulate Cortex; AMY, Amygdala; RSC, Retrosplenial Cortex; mPC, Medial Parietal Cortex; OPC; Occipito-parietal Cortex; and L/R, Left/Right Hemispheres.

Further supporting the effectiveness of the CERT intervention, brain imaging results showed significant changes in the rsFC (Table 2), overall, supporting the idea that the our ER training facilitated functional decoupling of bottom-up emotion and perception processing regions from regions of the DMN, along with enhanced functional coupling among top-down cognitive control regions and between regions of default-mode and control networks. First, for the subcortical seed regions, the AMY showed overall decreased rsFC with cortical regions involved in higher-level cognition and lower-level perception, following training (Figure 4). Specifically, across both groups, there was decreased rsFC between the left AMY seed and clusters in the cortical midline regions, including the medial frontal (mPFC/ACC) and parietal cortices. Interestingly, in the CERT group, the left AMY showed larger decrease in the rsFC with a cluster in the left visual cortex.

**Table 2:** Brain regions showing changes in functional connectivity following training.

Brain region	Side	BA	MNI coordinates			Peak *t*-value	Cluster size	
			*x*	*y*	*z*		
**L Amygdala**							
**Main Effects**							
↓ mPFC/ACC	R	32	15	39	−12	7.25	182
	R	10	14	41	−11	7.10	
	R	9	11	47	19	4.73	
↓ RSC/mPC	M	29	−6	−39	12	6.69	105
	M	23	−9	−54	12	5.05	
	M	31	−1	−64	27	4.07	
**Interaction Effects**							
↓ Precentral gyrus	M	4	0	−18	69	10.70	163
	L	5	−15	−44	85	6.69	
↓ Occipito-parietal Ctx.	L	19	−12	−81	45	7.43	106
	L	7	−18	−74	55	4.92	
**R Amygdala**							
**Main Effects**							
↓ RSC^*^	M	29	−3	−42	9	6.32	131
↓ Occipital Ctx.^*^	M	23	−6	−58	10	4.19	
↓ Cerebellum^*^			−1	−48	−15	4.52	
**L dlPFC**							
**Main Effects**							
↓ Lateral occipital Ctx.	L	18	−24	−93	0	7.77	113
↓ Occipital pole	L	17	−12	−100	−12	4.90	
**Interaction Effects**							
↑ dACC	M	32	9	18	39	7.55	128
↑ Medial frontal Ctx.	M	8	6	26	46	6.09	
	M	6	5	11	58	5.74	
↑ Inferior frontal Ctx.	R	44	52	15	3	5.19	80
**R dlPFC**							
**Interaction effects**							
↑ Middle frontal Ctx.	L	8	−42	12	45	8.79	72
↑ Medial occipital Ctx.	M	18	9	−87	−6	7.62	83
↑ Occipital pole	R	17	20	−96	−14	5.51	
**R vlPFC**							
**Main Effects**							
↑ Occipito-parietal Ctx.^*^	M	19/7	9	−75	39	7.43	186
↑ Occipital Pole^*^	M	18	12	−94	27	5.50	
↑ Occipital Ctx.^*^	R	18	15	−96	24	5.11	
**Interaction Effects**							
↑ Inferior Frontal Ctx.	R	9	39	12	24	7.00	92
↑ Occipito-temporal Ctx.	R	37	60	−57	0	7.36	109
↑ Occipito-temporal Ctx.	L	37	−63	−54	−9	6.86	74
**Medial PFC**							
**Main Effects**							
↑ Inferior parietal lobule	R	40	42	−45	57	6.47	97
↑ Superior parietal lobule	R	7	18	−66	55	5.83	
↑ Occipito-parietal Ctx.	M	19/7	−6	−81	39	7.90	76
	L	19	−18	−85	42	6.39	
**Interaction Effects**							
↑ Inferior parietal lobule	L	40	−42	−45	57	8.16	203

The table contains peak coordinates for clusters identified as showing significant changes in the resting-state functional connectivity (rsFC) across the cognitive-emotional regulation training (CERT) and psychosocial training (PSYCT) groups (i.e., Main Effects of Time) or stronger for the CERT group (i.e., Time × Group Interaction Effects). In the case of the main effects, up arrows indicate increases in the post-training rsFC and downward arrows indicate decreases in the post-training rsFC. In the case of the interaction effects, upward arrows indicate stronger rsFC increases in the CERT group than in the PSYCT group and downward arrows indicate stronger rsFC decreases in the CERT group than in the PSYCT group, following training. For main effects, the *t*-values represent Post vs. Pre differences, averaged across the CERT and PSYCT groups, and for interaction effects, *t*-values represent of Post vs. Pre differences between the two groups. Unless otherwise noted ^*^, regions listed were identified with a *p* < 0.005 threshold and FDR cluster-size corrected (*q*_FDR_ < 0.05).

* significant at a threshold of *p* < 0.01, *q*_FDR_ < 0.05.

BA, brodmann area; MNI, montreal neurological institute; Ctx., cortex; PFC, prefrontal cortex; mPFC, medial prefrontal frontal cortex; ACC, anterior cingulate cortex; RSC, retrosplenial cortex; mPC, medial parietal cortex; MFC, middle frontal cortex; dlPFC, dorsolateral prefrontal cortex; dACC, dorsal anterior cingulate cortex; IFC, inferior frontal cortex; vlPFC, ventrolateral prefrontal cortex; M, medial; and L/R, Left/right hemispheres.

**Figure 5:**
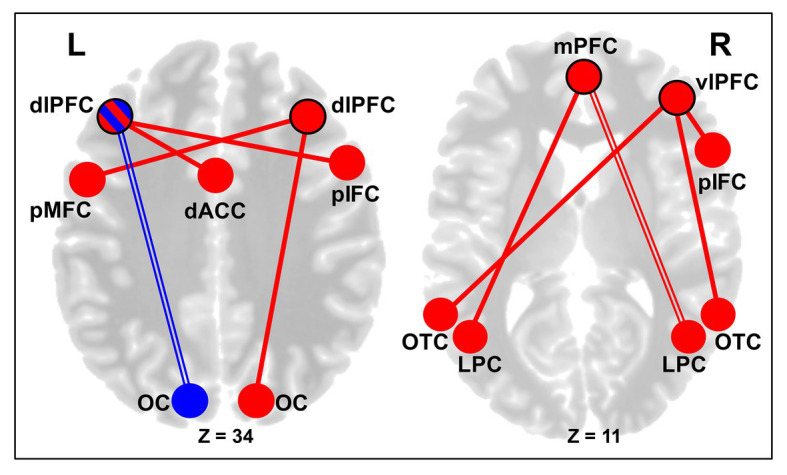
Changes in the rsFC of Control Regions following Training. Left panel: altered dorsolateral prefrontal cortex (dlPFC) rsFC following training. Subjects across both CERT and PSYCT groups showed reductions in rsFC between the left dlPFC seed and areas of the left visual cortex, following training (blue double line). CERT training was also associated with stronger increases in the rsFC between the left dlPFC seed and the dorsal anterior cingulate cortex (dACC), and between the left dlPFC seed and a posterior portion of the right inferior frontal cortex (red solid lines). Additionally, the CERT training was also associated with larger increases in the rsFC between the right dlPFC seed and areas of the right visual cortex, and between the right dlPFC seed and a posterior area of the left middle frontal gyrus (red solid lines). Right panel: increased ventrolateral prefrontal cortex (vlPFC) and mPFC rsFC following training. The CERT training was associated with stronger increases in the rsFC between the right vlPFC seed and a posterior area of the inferior frontal cortex (IFC; pIFC), as well as between the right vlPFC seed and the bilateral occipito-temporal cortex (OTC). Regarding the mPFC, there was increased post-training rsFC between the mPFC seed and the right LPC across both CERT and PSYCT groups (red double line), and the CERT training was also associated with larger increases in the rsFC between the mPFC seed and the left LPC (red solid line). Blue reflects decreases in functional connectivity, red reflects increases, and their combination (left dlPFC node) reflects both. Black node outlines indicate the seed regions. dlPFC, dorsolateral prefrontal cortex; MFC, middle frontal cortex; vlPFC, ventrolateral prefrontal cortex; IFC, inferior frontal cortex; mPFC, medial prefrontal cortex; dACC, dorsal anterior cingulate cortex; OC, occipital cortex; OTC, occipito-temporal cortex; LPC, lateral parietal cortex; and L/R, left/right hemispheres.

Second, for the cognitive control cortical regions, the left dlPFC showed decreased rsFC with a cluster in the left occipital pole, which was seen across both CERT and PSYCT groups. However, the left dlPFC showed larger increase in rsFC with a cluster in the cingulate/paracingulate cortex as well as with a cluster in the right posterior segment of the inferior frontal cortex (IFC), in the CERT group (Figure 5, left panel). Moreover, increased rsFC was also found between the right dlPFC and a cluster in the posterior part of the left middle frontal cortex, which was also larger, in the CERT group. Regarding the vlPFC seeds, there was increased rsFC between the right vlPFC seed and a cluster in the posterior segment of the right IFC, as well as clusters in the bilateral occipito-temporal cortex (OTC), all of which were larger in the CERT group. Finally, regarding the mPFC seed, there was increased rsFC with clusters in the right lateral parietal cortex (LPC), across both groups, and with a cluster in the left LPC, which was stronger in the CERT group (Figure 5, right panel).

Overall, these findings pinpoint training-dependent changes in bottom-up as well as top-down processes that are less driven by the emotional salience and basic perceptual processing, along with enhanced *in-tune* responses among cognitive and emotion control regions and across self-referential and cognitive control networks.

## Discussion

This report presents proof-of-principle evidence for the effectiveness of a novel cognitive-emotional training intervention targeting the acquisition of ER skills aimed at increasing resilience against emotional distress in military veterans. There were two main novel results: (1) behavioral results showed evidence for enhanced psychological well-being and executive function following training, reflected in increased GSE, PTG, and WM; (2) brain imaging results showed evidence of diminished bottom-up influences from emotional and perceptual brain regions, along with evidence of normalized rsFC in the large-scale functional networks following training, reflected in increased connectivity among cognitive and emotion control regions and across DMN and FPCN networks. These findings are discussed below.

### Enhanced Well-Being and Executive Function Following ER Training

First, CERT, but not PSYCT, was associated with increased GSE. GSE is not constrained to specific types of tasks or situations, but rather reflects more general beliefs in one’s competence to manage a broad range of tasks and challenges (Luszczynska et al., 2005). Therefore, successful performance of a range of behaviors and activities in a variety of situations is expected to build such a generalized sense of efficacy (Schwarzer and Luszczynska, 2007). The enhanced GSE as a result of engaging in the complex CERT is in line with previous intervention programs that have shown an efficacy-enhancing impact of a range of mastery experiences (Schunk, 1989; Bandura, 1997). Increased GSE is a very desirable outcome, given that SE is seen as the most crucial and proximal predictor of behavior (Bandura, 1997, 2002). SE has a self-regulatory function in dealing with stress and negative affect, being linked to better psychological adjustment, lower distress, fewer symptoms of burnout, and fewer symptoms of depression over time, along with reduced social anxiety, depression, and externalizing symptoms and better posttraumatic recovery (Brouwers and Tomic, 2000; Benight and Harper, 2002; Benight and Bandura, 2004; Bisschop et al., 2004; Gallagher et al., 2011, 2020; Singh and Bussey, 2011). It is assumed that SE impacts the appraisal and interpretation of stressful situations (transactional stress theory) and exerts beneficial effects through constructive regulation of motivational, affective, and decisional processes (Lazarus and Folkman, 1984; Bandura, 1997; Simmen-Janevska et al., 2012). This leads individuals with higher levels of self-efficacy to consider anxiety and stress symptoms as more controllable and temporary (Cervone, 2000; Leganger et al., 2000). We expect that, with a larger sample, the increased self-efficacy in our CERT group will also be associated with multiple benefits on well-being.

Second, both CERT and PSYCT were associated with increased PTG. One essential component of PTG is the ability to manage distressing emotions elicited by the traumatic event. The PTG model proposed by Tedeschi and Calhoun (2004) suggests that the process of rebuilding disrupted beliefs involves significant cognitive processing that allows engagement with trauma-related emotions and memories. Recent research examining the relation between ER and PTG suggests that strategies that involve engagement with the emotional stimuli, such as reappraisal, might influence PTG by helping individuals extract meaning from their traumatic experiences (Larsen and Berenbaum, 2015). Whereas reappraisal seems to be the choice for dealing with low-intensity situations, attentional deployment strategies are less cognitively demanding and seem to be preferred in high-intensity situations in individuals who had experienced a recent traumatic event (e.g., in the last 6 months, Strauss et al., 2016; Orejuela-Dávila et al., 2019). Both of our training programs involved significant cognitive processing. CERT, which offered our participants the flexibility to choose between FA and CR, allowed both engagement with diverse negative stimuli (pictures, memories, and worries) and disengagement of attention from the negative aspects of these stimuli, and facilitated PTG. The beneficial effect of ER training on PTG is in line with available evidence showing a positive correlation between self-reported engagement of adaptive ER strategies and PTG in undergraduate students (Thomas et al., 2019).

Third, participants in the CERT group also showed post-training improvements in WM, and these increases were observed in both Letter and Space tasks, while the PSYCT group participants did not show such increases. Individuals with emotional disorders such as anxiety and depression show a bias toward processing of negative emotional information (threat, sadness, etc.), leading to excessive rumination on past negative events and worry about the uncertainty of the future (for a review, see Mogg and Bradley, 2018). Our brain systems have a limited capacity, and thus when people get stuck on negative (and task-irrelevant) information, they have fewer resources to invest to complete the demands of the tasks at hand, shift attention, and process information efficiently (Derakshan and Eysenck, 2009). This gives rise to patterns of cognitive inflexibility (for a review, see Stange et al., 2017). When executive functions of WM become inefficient and rigid, people are more likely to experience interference and find it difficult to achieve their goals efficiently (Berggren and Derakshan, 2013).

Recent research suggests a strong link between WM, attentional control, and cognitive reappraisal (Shipstead et al., 2014). In fact, some authors argue that WM capacity refers specifically to attention control (Kane et al., 2001). Successful WM needs efficient use of attentional control to hold information temporarily and to manipulate the content in order to execute ER tasks, which require overriding habitual responses. Moreover, evidence from cross-sectional and training studies have established that WM ability and ER are connected, and that this connection is likely mediated by attention control (Schmeichel et al., 2008; McRae et al., 2012; Schweizer et al., 2013). The two ER strategies trained in the CERT group involve a number of mental operations, such as keeping in mind the specific ER strategy, monitoring/resolving the conflict between habitual and targeted reactions, selection among possible alternatives, and the modulation of behavior that, repeated over the course of the training, have helped participants improve their WM (Ochsner et al., 2012).

Finally, it should also be noted that an important aspect of the present training linked to the engagement of memory processes is not only related to WM *per se*, as discussed above, but also related to “*working with memory*.” This is because our ER training also affected the way participants encoded and retrieved emotional episodic memories. Indeed, there is evidence that both ER strategies affect emotional memory during both stages (Denkova et al., 2015; Dolcos et al., 2020a), with the overall tendency for reduction of the impact of emotion on memory (but see Dillon et al., 2007). In particular, by focusing away from the most emotional aspects of *external* stimuli, the engagement of FA is associated with reduced recollection of memory for emotional pictures (Dolcos et al., 2020a). Similarly, FA is also effective in decreasing the impact of recollected emotional autobiographical memories, both when retrieved in isolation and when retrieved as *internal* emotional distraction during an ongoing cognitive task (Denkova et al., 2015; Iordan et al., 2019). Hence, it is reasonable to expect that training participants to use ER strategies with external and internal stimuli have longer-lasting effects on the encoding and retrieval of memories for emotional events.

### Changes in Resting-State Functional Connectivity Following ER Training

The brain imaging findings from our pilot investigation provide further evidence for the effectiveness of the intervention in the CERT group. First, reduced functional connectivity between AMY and midline cortical structures (both frontal and parietal) is consistent with the idea of normalized bottom-up emotional influences on activity in DMN brain regions (Zilverstand et al., 2017). DMN has been linked to a range of self-referential processing (internal thought, memory retrieval, future planning, and emotion regulation), and hence reduced rsFC from a basic emotion processing region suggests diminished influences that would emotionally color the affective state of participants when turning their focus on the internal environment, which is typically associated with a negative emotional bias in emotional disturbances (Nolen-Hoeksema, 1991; Raichle et al., 2001; Fox et al., 2005; Schacter et al., 2007; Cooney et al., 2010; Denkova et al., 2015). Interestingly, these effects were observed across the participants from CERT and PSYCT groups, which suggests similar mechanisms of change in the expected direction. However, further supporting diminished default interactions between the AMY and perceptual areas, the reduced AMY-visual cortex rsFC was larger in the CERT group. This finding is consistent with the differences in training between the CERT and PSYCT groups. Whereas the PSYCT training primarily took the form of interpersonal communications about higher-level ideas (e.g., goals, values, and skills), the CERT training emphasized cognitive mechanisms (e.g., FA), including processing of visual stimuli, in combination with advanced cognitive processing (i.e., CR), meant to diminish the impact of emotional stimulation at different stages of emotion processing. Hence, reduced coupling between regions involved in bottom-up emotion signaling specifically observed in the CERT group suggests that this training equips participants with skills that reduce the impact of bottom-up emotional influences. This is particularly important in preparing them to respond quickly in emotionally intense situations, where more effortful emotion regulation might be difficult to deploy immediately (Sheppes et al., 2014; Orejuela-Dávila et al., 2019).

Turning to brain regions involved in cognitive/emotional control, reduced rsFC of the left dlPFC with the visual cortex is also consistent with diminished bottom-up influences from perceptual brain regions, a change that was also observed across the CERT and PSYCT groups. The overall greater increased connectivity of dlPFC ROIs, bilaterally, with vlPFC and dACC areas following the CERT intervention suggests strengthened default cross-talk among areas associated with cognitive/executive and affective control regions promoted by our ER training. This is important, given the central role of the dlPFC as part of the FPCN, which is involved in interfacing between focusing on internal and external stimulation, together with brain regions (vlPFC and ACC) circumscribed by other networks (salience and cingulo-opercular; Fuster, 1997; Smith and Jonides, 1999; Hopfinger et al., 2000; Corbetta and Shulman, 2002; Seeley et al., 2007; Dosenbach et al., 2008). This allows flexible behavior involving adaptive switches between paying attention to external stimulation and being aware of our internal states, needs, thoughts, and memories (Sridharan et al., 2008; Zabelina and Andrews-Hanna, 2016). Finally, increased rsFC connectivity among subregions of the right IFC (anterior and posterior), along with stronger increased connectivity between mPFC and lateral parietal cortical areas, in the CERT group, are also consistent with enhanced functional coupling among control brain regions and normalized cross-network interactions, respectively, promoted by the CERT training.

Interestingly, our expected increased connectivity between the AMY and ER brain regions was not confirmed. Our expectation was based on task-related evidence of increased functional coupling between basic emotion processing (AMY) and emotion control (lateral and medial PFC) regions, possibly indexing the need to regulate (Dolcos et al., 2006; Denkova et al., 2015). This is consistent with the idea that such coupling is necessary for the latter regions to exert control on the AMY response, when facing external or internal emotional challenges (Dolcos et al., 2006; Denkova et al., 2015). However, it may be the case that such couplings may only be transiently increased by current challenges and not necessarily also implemented in longer-term changes reflected by measures of rsFC. Further research is needed to clarify this matter.

### Caveats

A caveat of the current study is the limited sample, as there were only 19 participants in total. Future research should further confirm the proof-of-principle results of this pilot study in larger samples. Despite the smaller sample size, our findings showed strong improvements in post-traumatic growth, self-efficacy, and working memory, although there were no significant decreases in anxiety and depression as a result of the training. It is possible that changes in our primary outcomes of psychological distress have not been detected at the behavioral level due to the relatively small sample sizes of the groups in our pilot study. Nevertheless, some of the factors of behavior change, such as self-efficacy, might be more sensitive to the particular type of training offered by our interventions, and they may act as buffering mechanisms that contribute to the protection against symptoms of distress. Moreover, changes in the rsFC following both CERT and PSYCT training indicate a normalization of bottom-up emotional influences on activity in self-referential processing regions, suggesting that both types of training helped participants experience diminished negative affect when focusing on internal thoughts and worries. Second, the combination of FA and CR in the CERT was the most effective proof-of-concept strategy for testing our approach grounded in interactive person-situation-strategy processes and the emotion regulation choice framework (Sheppes et al., 2014). However, their relative contribution to the observed effects is not clear. Although it is reasonable to expect that FA contributed to improved WM and CR to the enhanced PTG, future research using multiple control groups is needed to disentangle the unique contribution of each of these strategies.

## Conclusion

Reduced ability to control emotional responses is a major marker of affective disturbances. Despite the small sample, the present pilot study provides proof-of-principle evidence for a sustainable cognitive emotion-regulation training intervention that goes beyond costly traditional models of therapeutic treatment by targeting the development of healthy and flexible ER skills in a sample of military veterans. Using a combination of behavioral and brain imaging methods, this study showed enhanced executive function and psychological well-being following training, reflected in increased working memory, post-traumatic growth, and general self-efficacy. Moreover, brain imaging results showed evidence of diminished bottom-up influences from emotional and perceptual brain regions, along with evidence of normalized functional connectivity in the large-scale functional networks following training, reflected in increased connectivity among cognitive and emotion control regions and across regions of self-referential and control networks. Overall, our results provide proof-of-concept evidence that resilience and well-being can be learned through ER training, and that training-related improvements manifested in both behavioral change and neuroplasticity can translate into real-life benefits.

## Data Availability Statement

The data sets analyzed for the current report are available upon requests made to the corresponding authors, pending approval from participants and the Institutional Review Board (IRB).

## Ethics Statement

The study was approved by the IRB office at the University of Illinois at Urbana-Champaign, and all participants provided written informed consent. The procedures used in this study adhere to the tenets of the Declaration of Helsinki.

## Author Contributions

SD and FD designed the study, with input from YH, CW, and HB. YH and CW collected the data. SD, FD, and YH planned the analytical approach, with input from HB. YH performed the analyses, with help from PB and KH and input from FD and SD. SD and FD wrote the first draft of the manuscript, and then revised it based on contributions from YH, PB, KH, and CW, and feedback from HB. All authors contributed to the article and approved the submitted version.

### Conflict of Interest

The authors declare that the research was conducted in the absence of any commercial or financial relationships that could be construed as a potential conflict of interest.

## Footnotes

^1^http://www.fil.ion.ucl.ac.uk/spm
